# Relationship between Rumen Microbial Differences and Phenotype Traits among Hu Sheep and Crossbred Offspring Sheep

**DOI:** 10.3390/ani14101509

**Published:** 2024-05-20

**Authors:** Haibo Wang, Jinshun Zhan, Haobin Jia, Haoyun Jiang, Yue Pan, Xiaojun Zhong, Shengguo Zhao, Junhong Huo

**Affiliations:** 1 College of Animal Science and Technology, Gansu Agricultural University, Lanzhou 730070, China; wanghaibo8815@163.com; 2 Institute of Animal Husbandry and Veterinary, Jiangxi Academy of Agricultural Science, Nanchang 330200, China; zhanjinshun1985@163.com (J.Z.); jiahaobin@jxaas.cn (H.J.); jianghaoyun1995@163.com (H.J.); py13782525871@163.com (Y.P.); zhongcaoyangchu@163.com (X.Z.); 3 College of Animal Science and Veterinary Medicine, Tianjin Agricultural University, Tianjin 300384, China

**Keywords:** rumen microbial, serum indicators, rumen enzyme activity, rumen fermentation, sheep

## Abstract

**Simple Summary:**

Simple Summary: The rumen is the most important digestive organ of ruminants, and its rich microbial co−evolved with the host to play a crucial role in immunomodulation, defense, digestive metabolism, animal productivity, and meat quality control. It also forms a complex regulatory network with host genes and phenotypes that are involved in maintaining organismal homeostasis. In this study, we compared the differences in rumen microorganisms between crossbred progeny of sheep and Hu sheep, identified key biomarkers, and analyzed their relationship with rumen function. The results showed that microbial diversity was significantly higher in crossbred progeny, which was able to alter the microbial community structure to regulate rumen fermentation, promote carbon homeostasis, and enhance amino acid metabolism. In addition, there were significant correlations between biomarkers and key traits. Therefore, we suggest that there is a link between sheep rumen microbes and differential traits in crossbred progeny, which provides new insights for sheep trait improvement.

**Abstract:**

This experiment was conducted to investigate the effect of three–way hybrid sheep and Hu sheep on serum indicators, rumen fermentation, rumen enzyme activity, and microorganisms in sheep. Healthy and similar birth weights from three groups (Hu, *n* = 11; Charolais × Australian White × Hu, CAH, *n* = 11; Charolais × Dorper × Hu, CDH, *n* = 11) were selected to be fed by the ewes until 45 days of age. Subsequently, they were weaned intensively and underwent short–term fattening for 3 months along with selected male lambs fed intensively. During this period, they were fed and watered ad libitum. Blood and rumen fluid were collected and analyzed for serum indicators and rumen fluid microorganisms, enzyme activity, and VFA, respectively, at the end of the fattening period. Compared with Hu lamb, the offspring of the three–way hybrid lamb showed significant improvements in body weight, serum lactate dehydrogenase, and creatinine content. However, there was no significant effect on serum immunity and antioxidant indices. In addition, the rumen fluid volatile fatty acid (VFA) molar concentration and microcrystalline cellulose and lipase content were significantly lower in the three–way hybrid lamb compared to Hu lamb, but β–glucosidase, amylase, pepsin, and VFA molar ratio were not significantly affected. Subsequently, 16S rRNA sequencing diversity analysis revealed that three–way hybrid lamb significantly increased rumen microbial ACE and Chao1 indices compared to Hu lamb. Meanwhile, the abundance of Verrucomicrobiota and Synergistota significantly increased at the phylum level. Correlation analysis showed that *Prevotella* had the highest proportion, while *Rikenellaceae_RC9_gut_group* correlated most closely with others genus. The microbial communities isovaleric acid molar concentration and proportion were strongly correlated. In addition, there were significant differences in correlations between microbial communities and isobutyric acid, butyric acid and valeric acid content, and their molar proportion, but they were not significantly correlated with digestive enzymes. From the functional enrichment analysis, it was found that hybrid progeny were mainly enriched in the pyruvate metabolism, microbial metabolism in diverse environments, carbon metabolism, and quorum sensing pathways. In contrast, the Hu sheep were primarily enriched in the cysteine and methionine, amino sugar and nucleotide sugar, and biosynthesis of secondary metabolite pathways. These results suggest that hybridization can play a role in regulating organismal metabolism and improve animal production performance by influencing the structure and characteristics of microbial communities.

## 1. Introduction

The sheep (*Ovis aries*) is an important livestock species that has been significant since the Neolithic period. Over time, various local breeds with unique characteristics have been developed through long–term natural and artificial selection [1]. Interestingly, sheep is known for its ability to convert plant feed (i.e., straw, hay, and grass) into meat, wool, skin, and milk for human needs [2,3]. The rumen is the most important digestive organ of ruminants, and the diverse microorganisms play a crucial role in feed conversion [4]. In addition, rumen microbes and hosts evolve synergistically during long–term adaptation to the environment. In this case, the host creates a suitable internal environment for the microbes, which metabolize to produce VFAs (volatile fatty acids) for use by the host. However, the structure of the rumen microbes also affects the host phenotype [5,6,7]. Notably, rumen microbes produce nutrients such as VFAs through fermentation, which can provide up to 70% of the host’s energy requirements. In particular, VFAs act as a bridge between microbiota and host interactions. VFAs can directly or indirectly participate in the regulation of organismal metabolism, mediate intestinal immune responses, and remodel gut microbial structure [8,9]. Furthermore, rumen microorganisms, as important regulators of ruminant growth performance, have significant heritability. They form a complex regulatory network with host genes and phenotypes, playing a crucial role in immune regulation, defense, digestion, and metabolism. In addition, they also contribute to the control of animal productivity and meat quality [4,10,11,12].

In production practice, researchers crossbreed introduced breeds with local breeds to enhance the productivity and meat quality of the offspring, or use this as a foundation for breeding new varieties [13,14,15]. In addition, when considering host genetics, the rumen microbial community can account for up to 20% of the effect on bodyweight in sheep [9]. Thus, a complex network of host genetics, rumen microbial communities, and sheep phenotypic traits exists. Previous studies have reported that the microorganisms in the rumen of ruminants (sheep [11,16,17,18,19], goat [20], cattle [21,22,23],dairy cows [24,25], yak [26]) are predominantly Bacteroidota and Firmicutes. Moreover, the microbial community varies among breeds and is influenced by host genetics, sex, environment, diet, age, and geographic range [9,27,28,29,30,31,32]. In addition, host genetics play a significant role in the formation of rumen microbial communities, including hybrid dominance resulting from the interaction of parental genes, which can influence rumen microbial composition [33,34].

The Hu sheep, a locally first–class protected breed in China, has garnered attention both domestically and internationally for its characteristics of fast early growth, four seasons of estrus, early sexual maturity, and high multiple litter yield [35]. Therefore, the Hu sheep is usually used as the mother parent to improve growth performance through cross improvement, breeding new strains and new varieties [36,37]. Through crossbreeding different sheep combinations, it was found that the progeny resulting from crossing exotic breeds with local sheep exhibited good production performance [13,14,15]. Our previous study found that 3–way crossbreeding significantly improved meat quality of lambs, such as increasing crude protein, essential amino acids, and non–essential amino acid content [38]. Therefore, we hypothesize that host genetics may play a role in regulating organismal metabolism and maintaining organismal growth and development by influencing the diversity and structure of rumen microbial communities. Meanwhile, based on the above hypothesis, a specific Charolais ram was used as a terminal ram to cross two different ewes with genetics: Australian White × Hu and Dorper × Hu to produce a Charolais × Australian White × Hu (CAH) and Charolais × Dorper × Hu (CDH) which were compared with pure native Hu lambs. Subsequently, this study was conducted with Hu, CAH, and CDH sheep under the same rearing environment and feeding conditions, excluding the influence of other factors. We investigated the variations in serum indicators, rumen fermentation, rumen enzyme activity, and microbial species structure between three–way hybrid sheep and Hu sheep. This result can provide new insights for regulating rumen microorganisms and trait improvement in sheep.

## 2. Materials and Methods

All experimental designs and feeding management involving animals were approved by the Institute of Animal Husbandry and Veterinary, Jiangxi Academy of Agricultural Sciences (2010–JAAS–XM–01). The experimental site is located in Ganzhou (Ganzhou Lvlinwan agriculture and animal husbandry Co., Ltd., Jiangxi, China) modernization company (129.10 m above sea level, 26.12° N latitude, 115.29° E longitude).

### 2.1. Experimental Animals

The healthy and similar birth weight male lambs of Hu (Hu × Hu), CAH (Charolais × Australian White × Hu) and CDH (Charolais × Dorper × Hu) were to be weaned centrally and uniformly after nursing with their mothers until 45 days of age. Subsequently, 11 male sheep of each breed were selected for uniform mixed feeding and short–term fattening for 3 months. The feeding management and immunization procedures were carried out according to the company’s regulations. Thirty–three lambs from the three treatment groups were kept in the same enclosure with water ad libitum and fed at 8:00 am and 17:00 pm throughout the experimental period. (The feed of the company’s total mixed ration composition was 19.50% peanut vine, 17.50% festuca arundinacea schreb, 3.00% garlic peel, 25.33% corn, 12.77% wheat bran, 15.00% soybean meal, 3% corn gluten meal, 0.30% NaCl, 0.60%,CaCO_3_, 3.00% premix, and the diet with digestible energy of 10.82 MJ/kg, crude protein of 16.95%, neutral detergent fiber of 27.68%, acid detergent fiber of 17.31%, calcium of 0.65%, phosphorus of 0.35%). 

### 2.2. Analysis of Body Size Indices

At 0 and 135 days of age, the body weights (BW) of all lambs were measured using calibrated electronic scales. Meantime, body size index (body height (BH), body length (BL), chest circumference (CC), and tube circumference (TC)) were measured by a soft ruler. Among them, all measurements were carried out in the morning before feeding.

### 2.3. Analysis of Serum Indicators

Before the end of the experiment, all experimental sheep had 5 milliliters of blood collected from the jugular vein blood with a vacuum blood collection tube before feeding (Jiangxi Jingzhi Technology Co., Ltd., Nanchang, China). The blood was kept at room temperature for 2 h, and then serum samples were collected by centrifuging in a low–speed centrifuge (TDL–80–2B; Anting Scientific Instrument Factory, Shanghai, China) at 3500 rpm for 10 min, followed by storing at −80 °C pending further analysis. Serum biochemical indicators, such as glutamic oxalacetic transaminase (GOT), glutamic pyruvic transaminase (GPT), urea nitrogen (UN), glucose (GLU), high density lipoprotein cholesterol (HDL–C), low density lipoprotein cholesterol (LDL–C), creatinine (CRE), triglyceride (TG), total cholesterol (T–CHO), creatine kinase (CK), phosphofructokinase (PFK) were determined by using the relevant kit provided by Nanjing Jiancheng Bioengineering Institute (Nanjing, China) according to the instructions. In addition, immunoglobulin A (IgA), immunoglobulin M (IgM), immunoglobulin G (IgG), malondialdehyde (MDA), total antioxidant capacity (T–AOC), catalase (CAT), glutathione peroxide (GSH–Px), and total superoxide dismutase (T–SOD) were determined by referring to the detailed instructions of the kit (Shanghai Kexing Biotechnology Co., Ltd., Shanghai, China).

### 2.4. Analysis of Rumen Fermentation Parameters and Enzyme Activities

Referring to the method described by Guo et al. [20], 50 mL of rumen fluid was collected from each sheep using a gastric tube rumen sampler prior to feeding, which was subsequently packaged in frozen storage tubes in liquid nitrogen for rapid freezing, and finally sent to the laboratory using liquid nitrogen to be stored at −80 °C for subsequent analysis. Acetic acid (AA), propionic acid (PA), isobutyric acid (IBA), butyric acid (BA), isovaleric acid (IVA), and valeric acid (VA) were determined by gas chromatograph (GC–7890B, Agilent Technologies), and the specific method used referred to the study of Liu [39] et al. Subsequently, the total volatile fatty acids (TVFAs), acetic acid/propionic acid (A/P), and VFA molar proportion (acetic acid (AAR), propionic acid (PAR), isobutyric acid (IBAR), butyric acid (BAR), isovaleric acid (IVAR), and valeric acid (VAR)) were calculated. Meanwhile, rumen fluid pHs were determined immediately using a portable pH meter (PHBJ–260F; INESA Scientific Instruments Co., LTD, Shanghai, China). Further, we evaluated microcrystalline cellulose (MCC), β–glucosidase (β–Glu), xylanase, lipase, amylase, carboxymethyl cellulose (CMC), and pepsin in rumen fluid with reference to the detailed instructions of the kit (Shanghai Kexing Biotechnology Co., Ltd., Shanghai, China).

### 2.5. DNA Extraction and Analysis of Bacterial Community in Rumen

The bacterial DNA was extracted from 33 rumen samples using the TGuide S96 Magnetic Stool DNA Kit (Tiangen Biotech (Beijing, China) Co., Ltd.). The hypervariable region V3–V4 of the bacterial 16S rRNA gene was amplified with primer pairs 338F: 5′–ACTCCTACGGGAGGCAGCA–3′ and 806R: 5′–GGACTACHVGGGTWTCTAAT–3′. The thermocycling profile used here was as follows: 95 °C for 5 min followed by 20 subsequent cycles of denaturation at 95 °C × 30 s, 50 °C × 30 s, then 72 °C × 40 s, and a final extension at 72 °C for 7 min. PCR products were checked on agarose gel and purified through the Omega DNA purification kit (Omega Inc., Norcross, GA, USA). The purified PCR products were collected, and the paired ends (2 × 250 bp) was performed on the Illumina Novaseq 6000 platform (Beijing Biomarker Technologies Co., Ltd., Beijing, China).

### 2.6. High–Throughput Sequencing Data Analysis

The raw data were first quality filtered using Trimmomatic [40] (version 0.33), then primer sequences were identified and removed using Cutadapt [41] (version 1.9.1), followed by splicing of double–ended reads and chimera removal using USEARCH [42] (version 10) (UCHIME [43], version 8.1), and finally, the analyzed sequences, often called ASVs (amplicon sequence variants), were denoised using the DADA2 [44] method in QIIME2 [45] (version 2020.6) on the basis of default parameters. Taxonomy annotation of the ASVs was performed based on the Naive Bayes classifier in QIIME2 [45] using the SILVA database [46] (release 138.1) with a confidence threshold of 70%.

### 2.7. Statistical Analysis

The sheep body size index (body weight, body height, body length, chest circumference and tube circumference) using repeated measures procedure for data analysis in SPSS software (version 26.0) (SPSS Inc., Chicago, IL, USA) was used to analyze. One–way ANOVA in SPSS software (version 26.0) (SPSS Inc., Chicago, IL, USA) was used to analyze the sheep serum (biochemical, immunological, antioxidant) indicators, rumen fluid enzyme activity, and VFA levels. Among them, Duncan’s method was used to make multiple comparisons when variance analysis had significant differences. The data were represented as the means ± SEMs. A *p* value less than 0.05 was considered significant. Alpha diversity index (Chao1, Ace, Shannon, Simpson) of the sample was evaluated using QIIME2 (https://qiime2.org, accessed on 6 June 2020) software. Beta diversity was determined using QIIME (binary Jaccard distance) to evaluate the degree of similarity of the microbial communities of different samples. Beta diversity was analyzed using principal coordinate analysis (PCoA), and non–metric multidimensional scaling (NMDS), and Anosim analysis allowed testing of whether there were significant differences in beta diversity between different groups of samples. Spearman’s rank correlation analysis was performed based on the abundance of each sample at the genus level as well as the variation, and data with correlations greater than 0.1 and *p* values less than 0.05 were screened to construct a correlation network. Furthermore, we employed linear discriminant analysis (LDA) effect size (LEfSe [47]) to test the significant taxonomic difference among groups. A logarithmic LDA score of 3.5 was set as the threshold for discriminative features. Sequence functional abundance was predicted using PICRUSt2 [48] (v2.2.0–b).

## 3. Results

### 3.1. Effect of Crossbreeding on Body Size Indices, Serum Indices, Rumen Enzyme Activity, and Fermentation in Progeny

#### 3.1.1. Effects of Crossbred Offspring on Body Size Indices in Sheep

Lamb body size indices were determined, and the results are shown in Table 1. The results showed that time significantly affected body weight, body height, body length, chest circumference, and tube circumference of lambs (*p* < 0.05). In addition, there were significant differences between treatment groups in body weight and tube circumference (*p* < 0.05), but no significant differences between body height, body length, and chest circumference (*p* > 0.05). Further interaction by time×group was found to affect significantly the body weight of the lambs (*p* < 0.05), but there was no significant effect on body height, body length, chest circumference, and tube circumference (*p* > 0.05). CAH and CDH lambs had significantly higher body weights than Hu lambs at 135 days (*p* < 0.05), but there were no significant differences in body weights between CAH and CDH (*p* > 0.05). However, body height, body length, chest circumference, and tube circumference did not show differences between H, CAH, and CDH lambs at 0 and 135 days, respectively (*p* > 0.05).

#### 3.1.2. The Effect of Crossbred Offspring on the Serum Biochemical Indices

Serum biochemical indices were determined, and the results are shown in Table 2. The results showed that the CAH lamb had significantly higher serum levels of LDH, UN, and CRE than the H lamb (*p* < 0.05), but there was no significant difference compared to the CDH lamb (*p* > 0.05). Meanwhile, the levels of LDH and CRE in the CDH lamb were significantly higher than those in the H lamb (*p* < 0.05). However, GOT, GPT, albumin, BCA protein, GLU, HDL–C, LDL–C, TG, T–CHO, CK, and PFK did not show differences between H, CAH, and CDH lambs (*p* > 0.05).

#### 3.1.3. The Effect of Crossbred Offspring on the Serum Immune and Antioxidant Indices

In Table 3, immune and antioxidant–related indices are shown in the serum of lambs. The results showed that immunoglobulin (IgA, IgM, and IgG) and antioxidant–related enzymes (MDA, T–AOC, CAT, GSH–Px, and T–SOD) in the serum of crossbred offspring (CAH and CDH) lambs did not differ from those of H lambs (*p* > 0.05).

#### 3.1.4. The Effect of Crossbred Offspring on the Rumen Digestive Enzyme Activities

To investigate the efficiency of ration utilization by lambs, the content of rumen digestive enzymes was assessed as shown in Table 4. The results demonstrated that the concentrations of MCC and lipase in the rumen of H lamb were significantly higher than those of CAH and CDH lambs (*p* < 0.05). In addition, there was no difference in the content of xylanase and CMC between H and CDH lambs (*p* > 0.05), but they were significantly higher than those of CAH lambs (*p* < 0.05). However, β–glucosidase, amylase, and pepsin did not differ between crossbred offspring (CAH and CDH) and H lambs (*p* > 0.05).

#### 3.1.5. The Effect of Crossbred Offspring on the Rumen Fermentation Parameters

Herein, the fermentation parameters of lamb rumen were evaluated and are presented in Table 5. The results showed that rumen pH was significantly higher in the CAH and CDH compared to the H lamb, while acetic acid, propionic acid, butyric acid, valeric acid, and TVFA molar concentration were significantly lower (*p* < 0.05). However, there was no significant difference in the crossbred progeny CAH and CDH lambs (*p* > 0.05). Meanwhile, isovaleric acid, acetic acid/propionic acid (A/P), and VFA molar proportion (acetic acid, propionic acid, butyric acid, isovaleric acid, and valeric acid) were not significantly different between H and crossbred (CAH and CDH) offspring (*p* > 0.05). In addition, the rumen isobutyric acid content was significantly higher in H lambs compared to CAH lambs (*p* < 0.05), but it was not significantly different from CDH lambs (*p* > 0.05). However, it is noteworthy that the isobutyric acid molar proportion was significantly higher in the rumen of lambs of crossbred offspring (CAH and CDH) compared to H lambs (*p* < 0.05)

#### 3.1.6. Relationship between Physiological Metabolic Indices and Economic Traits in Lambs

**Figure 1:**
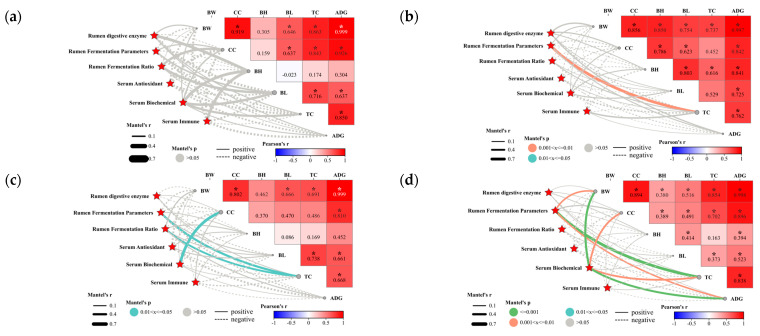
We analyze the relationship between physiological metabolism and growth traits in Hu (**a**), CAH (**b**), CDH (**c**), and lamb herd (Hu, CAH, and CDH date as a whole) (**d**). Pairwise comparisons of growth factors (BW, CC, et al.) are shown Figure 1. Spearman’s correlation coefficient is indicated by the color gradient. Edge width corresponds to the Mantel’s r statistic for the corresponding distance correlations, and edge color denotes the statistical significance based on permutations. The type of line indicates positive or negative correlation. In the heat map, * indicates significant correlation and values indicate correlation coefficients.

Correlation of rumen digestive enzyme activities, rumen fermentation parameters, rumen fermentation ratios, serum biochemical, immune, and antioxidant properties with lamb growth traits were evaluated and are presented in Figure 1. Overall, the body size indicators BW, CC, BH, BL, and TC of lambs showed a positive correlation with each other. Meanwhile, BW was significantly positively correlated with CC, BL, and TC. BW showed the strongest positive correlation with CC (Figure 1). The results of Mantel’s analysis of the treatment groups indicated that there was no significant correlation between any of the parameters and body size in the H group (Figure 1a). In the CAH group, only rumen fermentation parameters were significantly and positively correlated with TC (Figure 1b). In the CDH group, TC was significantly positively correlated with rumen fermentation parameters and rate, of which blood biochemistry was significantly positively correlated with CC (Figure 1c). Crossbreeding significantly influenced the correlation between serum biochemistry, rumen fermentation parameters, and lamb body size indices (Figure 1d).

### 3.2. Effect of Hybridization on the Composition and Function of the Rumen Microbial Community of Progeny

#### 3.2.1. Sequencing of Rumen Microbiota

Sequencing of 16S rRNA produced 2,639,659 pairs of reads from 33 samples. Subsequently, a total of 2,632,626 clean reads were generated by quality control and splicing of double–ended reads. At least 79,525 clean reads were generated in each sample, with an average of 79,777 clean reads (Supplementary Table S1). DADA2 method in QIIME2 (https://qiime2.org, accessed on 6 June 2020) being applied to denoise sequences, generating ASVs. Those sequences were assigned to 17,060 ASVs, of which 1153 ASVs were common among all groups (Supplementary Figure S1a). The dilution curve results showed that as the number of sequencing reads increased, the curve transitioned from a steep incline to a more gradual plateau, suggesting that the sequencing coverage had reached saturation (Supplementary Figure S1b).

#### 3.2.2. Diversity Analysis

**Figure 2:**
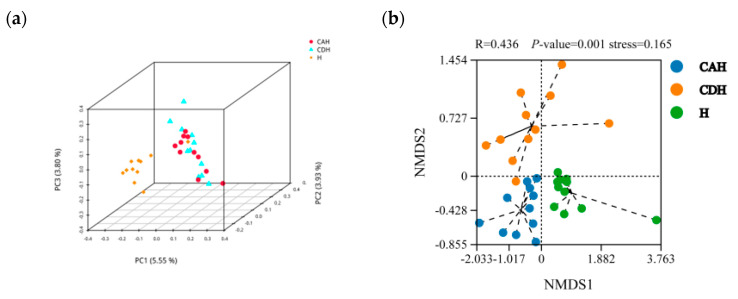
Beta diversity Composition comparison of rumen microbiota in the H, CAH, and CDH groups. (**a**) Principal coordinate analysis (PCoA), The coordinates indicate the selected primary coordinate component and the percentages indicate the contribution of the primary coordinate component to the difference in sample composition; (**b**) Non–MetricMulti–Dimensional Scaling (NMDS); stress less than 0.2 indicates that the NMDS analysis has some reliability. In this case, the closer the R–value is to 1, the greater is the between–group difference compared to the within–group difference, while *p* value less than 0.05 indicates that the between–group difference is significant. The closer the samples are on the coordinate graph, the higher is the similarity.

The rumen microorganisms’ alpha and beta diversity estimation of H, CAH, and CDH sheep are shown in Table 6 and Figure 2, respectively. Analysis of Table 6 reveals that H lamb is significantly lower than CAH and CDH lamb on both the ACE and Chao1 indices (*p* < 0.05), but it is not significantly different on either the Simpson or Shannon indices (*p* > 0.05). However, both CAH and CDH lamb were not significantly different in alpha diversity (*p* > 0.05). PCoA analysis was performed using the binary Jaccard method (Figure 2a). Among them, the closer the samples are on the coordinate diagram, the more similar they are. In the axes, PC1, PC2, and PC3, respectively, represent the contribution values of the first, second, and third principal components to the sample difference, which are 5.55%, 3.93%, and 3.80%. Furthermore, based on the NMDS analysis, the results show that the stress value of 0.165 is less than 0.2, indicating that the model has a certain level of reliability (Figure 2b). Meanwhile, in the analysis of similarity (ANOSIM), we can observe that R = 0.436, *p* = 0.001, which indicates significant differences between groups (Figure 2b). In conclusion, the rumen microbial diversity of H lamb was significantly different from that of CAH and CDH lambs.

#### 3.2.3. Composition of the Rumen Microbiota

**Figure 3:**
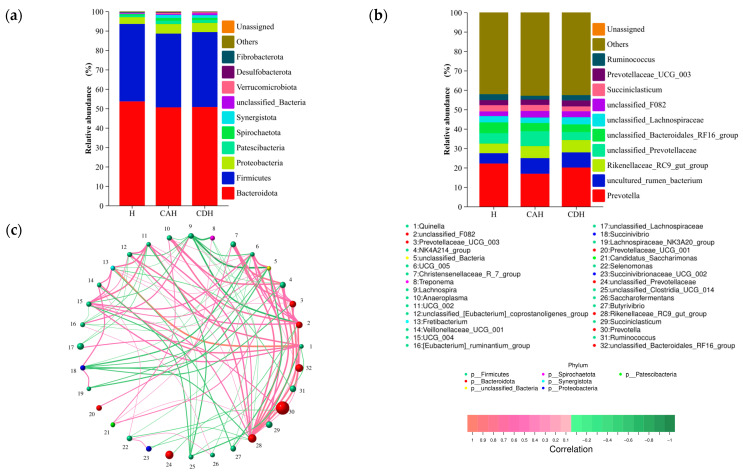
Stacked histograms of the relative abundance of the rumen microbiota of the H, CAH, and CDH groups at the phylum (**a**) and genus (**b**) levels of the Top 10, and correlation network plots for genus level abundances above 0.50% (**c**). The horizontal coordinate is the name of the grouping, the vertical coordinate is the proportion of the species in that sample, different colored bars represent different species, and the length of the bar represents the size of the proportion of that species. The color of the dots in the network diagram represents the phylum to which they belong, and the size indicates the abundance. Meanwhile, the color of the concatenated line indicates positive (red) or negative (green) correlation, and the thickness indicates the correlation size.

We analyzed the relative abundance of microorganisms annotated as ASVs at the phylum and genus levels. A total of 22 phyla and 346 genus were identified in the rumen microorganisms. At the phylum level, Bacteroidota is the most abundant, accounting for 53.62%, 50.57%, and 50.73% in the H, CAH, and CDH lamb groups, respectively (Figure 3a). Firmicutes (H: 39.93%, CAH: 37.80%, CDH: 38.62%) and Proteobacteria (H: 3.52%, CAH: 4.92%, CDH: 4.75%), respectively, were the second and third most abundant phyla based on 16S rRNA sequencing (supplementary Table S2). At the genus level, we found that the top five dominant bacteria ranked in H, CAH, and CDH were all *Prevotella*, *uncultured_rumen_bacterium*, *Rikenellaceae_RC9_gut_group*, *unclassified_Prevotellaceae*, and *unclassified_Bacteroidales_RF16_group* (supplementary Table S3). Subsequently, we plotted species distribution histograms for the 10 phyla (a) and genus (b) with the highest abundance, as shown in Figure 3. At the phylum level, Verrucomicrobiota and Synergistota had a significantly greater relative abundance in CAH and CDH than in H lambs (*p* < 0.05), but there was no significant difference in between CAH and CDH lambs (Figure 3a) (*p* > 0.05). At the genus level, *uncultured_rumen_bacterium* had a significantly greater relative abundance between CAH and CDH than in H lamb (*p* < 0.05), but there was no significant difference in CAH and CDH lambs (*p* > 0.05). Dominant microbial, including *Prevotella*, *Rikenellaceae_RC9_gut_group*, *unclassified_Prevotellaceae*, and *unclassified_Bacteroidales_RF16_group* showed no significant difference in the relative abundance of these dominant bacteria among groups H, CAH, and CDH lambs (Figure 3b). Meanwhile, by constructing a network diagram of genus level groups with abundances greater than 0.50%, it was found that *Prevotella* accounted for the highest percentage, while *Rikenellaceae_RC9_gut_group* was most closely correlated with other genus (Figure 3c).

#### 3.2.4. Microbial Composition Drives Rumen Homeostasis

**Figure 4:**
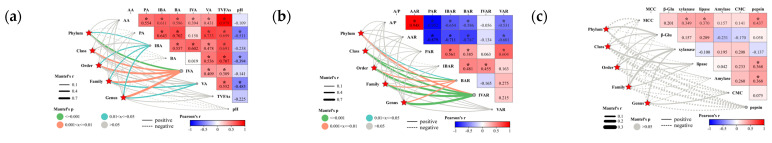
We analyzed the relationship between rumen microbial communities and rumen volatile fatty acid composition (**a**), VFA molar proportion (**b**) and rumen digestive enzyme activities (**c**) in lambs. Pairwise comparisons of rumen fermentation parameters (AA, PA, IBA, BA, IVA, VA, TVFAs, and pH.), molar proportion (A/P, AAR, PAR, IBAR, BAR, IVAR, VAR) and digestive enzyme activities (MMC, β–Glu, xylanase, lipase, amylase, CMC, pepsin) are shown Figure 4. Spearman’s correlation coefficient is indicated by the color gradient. Edge width corresponds to the Mantel’s r statistic for the corresponding distance correlations, and edge color denotes the statistical significance based on permutations. In the heat map, * indicates significant correlation and values indicate correlation coefficients.

Correlations between rumen microbial and rumen fermentation parameters, as well as fermentation ratios of lambs, were evaluated, as shown in Figure 4. In general, the pH of lamb rumen was negatively correlated with the content of VFA and showed a significant negative correlation with PA, BA, and VA (Figure 4a). In addition, AA, PA, IBA, BA, IVA, and TVFAs exhibited significant positive correlations when compared with each other. Microbial communities significantly influenced the levels of IBA, IVA, and VA, with the strongest correlation observed with IVA (Figure 4a). Further analysis of the percentage of VFA revealed significant negative correlations between both AAR and A/P with PAR, IBAR, BAR, and VAR. However, AAR and A/P exhibited a significant positive correlation (Figure 4b). In addition, PAR, IBAR, BAR, IVAR, and VAR showed overall positive correlations with each other (Figure 4b). Meanwhile, microbial communities were able to significantly influence BAR and IVAR, with the strongest association found being with IVAR (Figure 4b). However, there was no significant correlation between microbial composition and rumen enzyme activities (Figure 4c). In addition, there was an overall positive correlation between rumen enzyme activities (Figure 4c).

#### 3.2.5. Relationship between Biomarkers and Sheep Rumen Parameters

**Figure 5:**
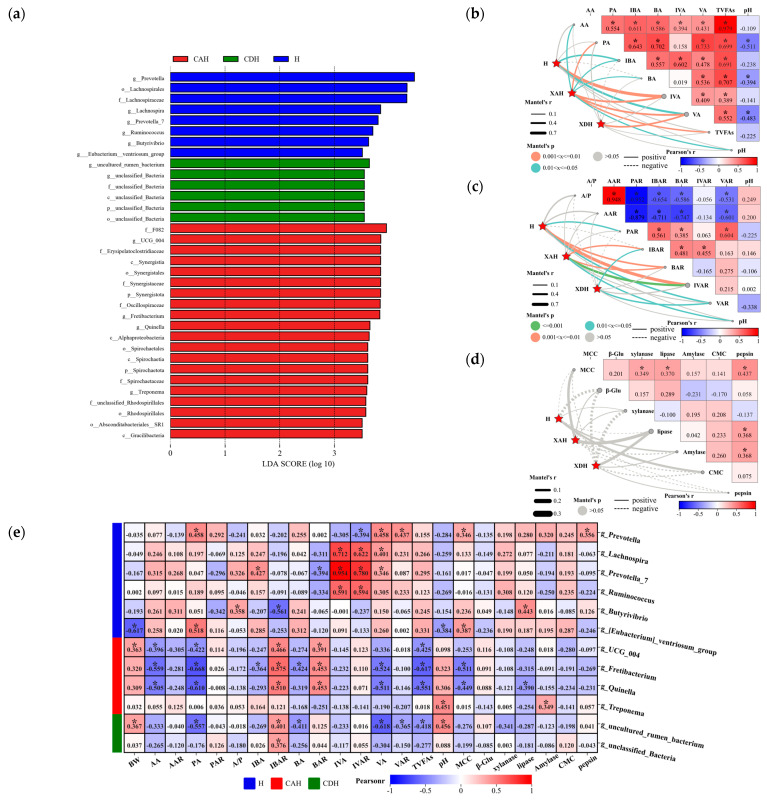
Biomarkers and sheep rumen parameters evaluated for analysis. Linear discriminant analysis effect size (LEfSe) analysis (**a**) and genus level biomarkers and rumen volatile fatty acid composition (**b**) and VFA molar proportion (**c**) in lambs. Pairwise comparisons of rumen fermentation parameters (AA, PA, IBA, BA, IVA, VA, TFAs, and pH) and VFA molar proportion (A/P, AAR, PAR, IBAR, BAR, IVAR, VAR, and pH) are shown Figure 5. Spearman’s correlation coefficient is indicated by the color gradient. Edge width corresponds to the Mantel’s r statistic for the corresponding distance correlations, and edge color denotes the statistical significance based on permutations. In addition, we performed correlation analysis of differential biomarkers with phenotypic indicators (**d**). In the heat map, * indicates significant correlation and values indicate correlation coefficients. Correlation analysis between genus level biomarkers and sheep body weight (BW), rumen VFA molar proportion, VFA molar concentration, and digestive enzyme activities (**e**).

LEfSe was used to analyze biomarkers in the rumen of crossbred offspring lambs, with an LDA score of 3.5. The analysis revealed 34 statistically different biomarkers in the rumen fluid of lambs from various crossbreeding offspring, including 8 in H, 20 in CAH, and 6 in CDH. At the genus level, Hu sheep were included in *Prevotella*, *Lachnospira*, *Prevotella_7*, *Ruminococcus*, *Butyrivibrio*, and *[Eubacterium]_ventriosum_group* with six biomarkers. The four biomarkers in CAH sheep were *UCG_004*, *Fretibacterium*, *Quinella*, and *Treponema*. The two biomarkers in CDH were *uncultured_rumen_bacterium* and *unclassified_Bacteria* (Figure 5a). To gain more insight into the effects of biomarkers on rumen parameters, we correlated biomarkers obtained with rumen fermentation parameters (Figure 5b), molar proportion (Figure 5c), and rumen digestive enzyme activities (Figure 5d). Overall, biomarkers were able to influence rumen fermentation parameters (AA, PA, IBA, BA, IVA, VA, TVFAs, and pH) to varying degrees, but the association was stronger with IVA and VA (Figure 5b). In addition, it was also able to significantly influence VFA molar proportion (PAR, IBAR, BAR, IVAR, and VAR), with a more pronounced effect on IVAR (Figure 5c). In addition, we found no significant correlation between biomarkers for A/P and AAR (Figure 5c). Meanwhile, rumen pH showed a significant negative correlation with PA, BA, and VA (Figure 5b), but it was not significantly correlated with A/P, AAR, PAR, IBAR, BAR, IVAR, and VAR (Figure 5c). In addition, biomarkers were not significantly correlated with rumen enzyme activity (Figure 5d). Biomarkers overall were not significantly correlated with BW. BW was only negatively correlated with the *[Eubacterium]_ventriosum_group*, but it was significantly positively correlated with *UCG_004* and *uncultured_rumen_bacterium* (Figure 5e). The combined analysis revealed significant correlations between biomarkers and rumen fermentation parameters. The analysis revealed that the correlations of the biomarkers with AA and VA and their molar proportion were similar. However, they differed significantly from PA, IBA, and BA correlations and their molar proportion. In addition, the correlation of the biomarker with TVFAs and its correlation with AA were similar, but the correlation with pH was significantly opposite (Figure 5e). Meanwhile, *UCG_004*, *Fretibacterium*, and *Quinella* were significantly positively correlated with IBAR and BAR, but they were significantly negatively correlated with AA, PA, and TVFAs, and *Fretibacterium* was negatively correlated with IBA, BA, and VA. Moreover, *Butyrivibrio* was significantly positively correlated with A/P and significantly negatively correlated with IBAR. In addition, *Lachnospira*, *Prevotella_7*, and *Ruminococcus* showed a significant positive correlation with IVA and IVAR. *Prevotella* showed a significant positive correlation with PA, VA, and VAR, but it showed significant negative correlation with IVAR. *Prevotella_7* showed significant negative correlation with BAR, but it also showed significant positive correlation with IBA and VA. In addition, *Lachnospira* showed significant positive correlation with VA. In general, the biomarkers were largely not significantly correlated with rumen MCC, β–glucosidase, xylanase, lipase, amylase, CMC, and pepsin (Figure 5e). Our analysis revealed that MCC was significantly positively correlated with *Prevotella*, *[Eubacterium]_ventriosum_group*, but it was significantly negatively correlated with *Fretibacterium* and *Quinella*. In addition, lipase was significantly positively correlated with *Butyrivibrio* but significantly negatively correlated with *Quinella*. Meanwhile, amylase and pepsin were positively correlated with *Treponema* and *Prevotella*, respectively (Figure 5e).

#### 3.2.6. Functional Prediction of Rumen Microbial in H, CAH and CDH

To predict rumen microbial function in H, CAH, and CDH, we performed PICRUSt2 using the Kyoto Encyclopedia of Genes and Genomes database (KEGG). PICRUSt2 prediction results of the H, CAH, and CDH groups showed that the main enrichment was in the metabolic pathway, and accounted for more than 71.97%, 71.85%, and 71.79%, respectively, including global and overview maps, carbohydrate metabolism, amino acid metabolism, metabolism of cofactors and vitamins, energy metabolism, and nucleotide metabolism. Meanwhile, microbiome functional enrichment analysis at Class 2 level top 10 showed that CAH amino acid metabolic pathway was significantly higher than Hu. Further analysis of the differences among the top 20 functional abundance pathways at the Class 3 level showed that there were significant differences in amino acid metabolism (cysteine and methionine metabolism), carbohydrate metabolism (amino sugar and nucleotide sugar metabolism, pyruvate metabolism), global and overview maps (biosynthesis of secondary metabolites, microbial metabolism in diverse environments, carbon metabolism), and cellular community prokaryotes (quorum sensing) pathways between Hu and CAH (*p* < 0.05). Interestingly, the microbial functional pathways in H versus CDH and CAH versus CDH were similar between the top 20, suggesting that rumen microbes share a common primary function and generate energy for host growth and development through microbial metabolism (Table 7). At present, the functional enrichment is only predictive, and the specific functional pathways need to be further investigated.

## 4. Discussion

### 4.1. Effect of Crossbreeding on Body Size Indices, Serum Indices, Rumen Enzyme Activity, and Fermentation in Progeny

Crossbreeding can make use of heterosis to make the offspring inherit the good characteristics of different varieties, and improve the growth performance of livestock and poultry. Stature (body size and body weight) is recognized as a highly heritable trait in mammals, which consists of many polymorphisms of small effect [49,50]. Meanwhile, body weight is an economically important property in sheep production and is influenced by host genetics and environmental factors, as well as genetics–environmental interactions such as rumen microecology. Our study found an overall significant positive correlation between weight and body size indicators, with the largest correlation coefficient being found between weight and chest circumference. The results of the present study are similar to those of previous studies, further confirming the developmental advantages of crossbred lambs [13,14,15]. The results of Wang et al. [9] proved that host genetics explained 39% of the total phenotypic variation, and the rumen microbial community had a 20% effect on sheep body weight, taking into account host genetics. In practice, improvement of genetic traits by direct selection is hard and time–consuming, while significant genetic gains can be achieved through crossbreeding using either local or exotic germplasm or using genomic introgression of genes with major effects [51]. Today, most breeding programs for higher prolificacy are based on traditional means of crossbreeding and within–cross or within–breed selection, including production and use of F1 crosses, production of three–way crosses, or development of composite sheep breeds [52]. This study found that utilizing Charolais as a terminal crossbreed significantly increased the body weight of crossbred lambs as compared to Hu lambs, which may be due to the fact that CAH and CDH lambs have inherited the excellent growth and developmental performance of their father, Charolais sheep. This result is consistent with previous studies that found that crossbreeding offspring of different breeds (Dorper × Santa Inês [13] Southdown × Hu [14], and Dorper × Sarda [15]) could improve the growth and development of lambs. Kong et al. [14] studies have demonstrated that amino acid metabolism plays an important role in the regulation of muscle development in crossbred sheep, especially arginine and proline metabolism, histidine metabolism, and tryptophan metabolism. Therefore, we found that crossbred offspring were improved compared to Hu lambs by enrichment analysis of rumen microbial functions in amino acid metabolism, especially arginine and proline metabolism, histidine metabolism, and tryptophan metabolism. Our research results are similar with the results of Kong et al. [14]. Therefore, we hypothesized that the use of Charolais as a terminal sire could influence the gastrointestinal microbial community of lambs in crossbred offspring, thereby altering the lamb phenotype.

In order to further investigate the potential effects of crossbreeding on lamb phenotypes, we analyzed the serum biochemical, immunological, and antioxidant parameters, rumen digestive enzyme activity, and rumen fermentation parameters of the lambs. We found that crossbreeding had essentially no significant effect on immunoglobulin (IgA, IgM, and IgG), antioxidant indices (MDA, T–AOC, CAT, GSH–Px, and T–SOD), and biochemical properties (GOT, GPT, albumin, BCA protein, GLU, HDL–C, LDL–C, TG, T–CHO, CK, and PFK) in the serum of lambs, and only significantly increased the activity of LDH, and the content of UN and CRE. However, the hybrid progeny significantly affected rumen enzyme activity and rumen fermentation parameters. It was found that CAH and CDH rumen MCC, lipase, AA, PA, IBA, BA, VA, and TVFAs molar concentration were significantly reduced, and pH was significantly increased. However, the crosses showed essentially no significant changes in VFA molar proportion. Meanwhile, rumen pH showed a significant negative correlation with the molar concentration of VFA (PA, VA, and BA), but a lower correlation with the molar concentration of VFA. Therefore, hybridization during this experiment mainly altered rumen digestive enzyme activity and VFA molar concentration. Studies have found that when VFA molar concentration increased, it was able to increase pancreatic sensitivity, leading to increased insulin secretion, increased fat oxidation, and decreased fat deposition and body weight [53]. In addition, when VFA concentration increases, it can increase free fatty acid receptor 2 activation, promote peptide YY and glucagon–like–peptides 1 secretion, increase anorexigenic signaling and decrease orexigenic signaling, affect food intake, lead to negative energy balance, and negatively regulate feed intake [54]. In summary, when the concentration of VFA in the body is high before feeding, it may affect the appetite of lambs and is not conducive to the growth and development of lambs. Meantime, in this experiment, it was found that the concentration of VFA in Hu lamb was significantly higher than that of CAH and CDH before fasting and the body weight was reduced compared to them. This result is consistent with the above theory [53] and further proves that there is a difference in the utilization efficiency of VFA between the organisms of Hu sheep and crossbred offspring. Interestingly, the relationship between serum indices and rumen parameters with body weight and body size differed between crossbred offspring and Hu sheep. Therefore, we speculate that crosses may influence offspring phenotypes by altering the rumen microbial community in the offspring lambs, further influencing rumen fermentation [21,55,56]. Meanwhile, the experimental results showed that the microbial and rumen fermentation parameters were similar in the crossbred offspring (CAH and CDH) of the same terminal sire and the same feeding management, but the crossbred offspring were significantly different from the purebred Hu sheep. In this regard, rumen microorganisms collectively ferment and degrade diets into VFAs and other nutrients that meet 70% of the energy requirements of ruminants [17,57]. The results showed that the molar concentrations of VFAs showed significant positive correlation with each other. Meanwhile, AAR was significantly positively correlated with A/P, but AAR and A/P were inversely correlated with the percentage of other VFAs. Interestingly, the results found in the present study indicated that VFA content (AA, PA, IBA, BA, IVAR, and TVFAs) in rumen fluid of crossbred offspring was significantly reduced, but there was no significant effect on A/P and VFA molar proportion (AAR, PAR, BAR, VA, and VAR). We speculate that this phenomenon may be related to the findings in the following study that the hybrid progeny improved the conversion efficiency of rumen VFAs by increasing the enrichment of rumen microbial functions in the carbon metabolism, and pyruvate metabolism pathways, and that the host maintains the homeostasis of the internal environment of the rumen through its own metabolism regulation to ensure the balance of the VFA molar proportion in rumen [58]. In summary, we found that CAH and CDH from the same terminal parent were phenotypically similar but significantly different from Hu lambs. This may be a systematic effect of the terminal sire on the germline through microorganisms, and consequently on the F1 offspring, which needs to be further explored [59].

### 4.2. Effect of Hybridization on the Composition and Function of the Rumen Microbial Community of Progeny

The gut microbiota is appreciated to play a principal role in integrating environmental signals into host responses. Rumen microbes are closely related to host traits [5,9], including involvement in fat deposition [18], body weight [9], feed efficiency [12,60], marbling grade [11], and more. Meanwhile, rumen microbes of ruminants are structurally similar [61]. In addition, rumen microbial communities are susceptible to host genetics, gender, age, environmental, diet, geographical range, and other factors [9,27,28,29,30,31,32]. However, when maintaining a high degree of consistency in external conditions, we hypothesized that host genetics may play a specific role in the formation of rumen microbes. In addition, related research reports have demonstrated that hybridization results from parental gene interactions and that different gene expression programs may affect rumen microflora [33,34]. In this study, we first evaluated the α and β diversity of rumen microorganisms. Our results showed that the ACE index and Chao1 index were significantly higher in CAH and CDH than in Hu sheep, but the Simpson and Shannon indices had no significant effect. Subsequently, we further analyzed PCoA and NMDS and found significant differences between Hu, CAH, and CDH. However, the diversity of rumen microorganisms in the CAH and CDH groups was more similar in the progeny of crosses from the same terminal sire. This result further validates the above findings, and the diversity of the rumen ecosystem is more conducive to the maintenance of rumen homeostasis and resistance to environmental changes [62]. This result suggests that terminal parent crosses alter the diversity of rumen microorganisms, and it may be more beneficial to maintain rumen homeostasis and resist environmental changes. Similar results were obtained in cattle [21,63], deer [64], goat [65], and sheep [17]. On average, we observed that the dominant bacteria in sheep is Bacteroidota and Firmicutes, which is consistent with previous ruminant (sheep [11,16,17,18,19], goat [20], cattle [21,22,23], dairy cows [24,25], and yak [26]) studies. Bacteroidota and Firmicutes are jointly involved in feed digestion and absorption, energy metabolism, and resistance to invasion by exogenous pathogens, so they may be associated with improved body weight in offspring [12,66,67]. Previous studies suggested that high Firmicutes and low Bacteroidetes populations indicated a high energy–harvesting ability of the host, and the reduction of Firmicutes/Bacteroidetes (F/B) was strongly associated with body weight loss [68], feed efficiency reduction [60], and dairy cows producing less milk fat [69]. This experimental study found that the rumen F/B of the crosses was higher than that of the Hu sheep. Meanwhile, during rumen fermentation, Bacteroidota, Firmicutes, Verrucomicrobiota, and Fibrobacterota were enriched for genes associated with the degradation of lignocellulosic polymers and the fermentation of degradation products into VFAs [70]. Monofluoroacetate is a potent toxin that blocks ATP production via the Krebs cycle and causes acute toxicity in ruminants consuming monofluoroacetate–containing plants [71]. Compared with aerobic degradation of monofluoroacetate, rumen Synergistota can utilize amino acids as substrates, and under anaerobic conditions is capable of producing fluoride and acetate from monofluoroacetate as the end–products of dehalorespiration [72,73]. It indicates that the relative abundance of Verrucomicrobiota and Synergistota was significantly increased in the hybrid progeny, which contributes to the enhancement of the organism’s resilience, the improvement of cellulose degradation, and the conversion into VFA to be utilized by the organism. At the genus level, we found the dominant bacteria *Prevotella*, *uncultured_rumen_bacterium*, and *Rikenellaceae_RC9_gut_group.* Of these, *Prevotella* had the highest proportion, while *Rikenellaceae_RC9_gut_group* was most closely related to the other genera. This is in agreement with previous studies that found that the predominance in the rumen of sheep was all *Prevotella* [11,17,74]. The high abundance of *Prevotella* is associated with a healthy microbial community and serves as the core of carbohydrate and hydrogen metabolism. One of its fermentation products is propionic acid, the most important substrate for gluconeogenesis in ruminant liver, which in turn competes with methanogenic and archaebacterial bacteria for hydrogen utilization, and whose abundance is negatively correlated with methane emissions [75,76]. While dominant bacteria play important roles in physiological functions, even more noteworthy are low–abundance bacteria, which, despite their lower abundance, have much higher taxa numbers and diversity than high–abundance bacteria in the host and may play key roles in various aspects of host physiology. Correlation analysis was utilized to find significant correlations between numerous low abundance bacterial genera and other genera. In addition, these microorganisms establish a reciprocal relationship with the host and can influence a wide range of host phenotypes, including immune activation, protection against pathogenic infections, and they also interact with other commensal bacteria in their ecological niche [77].

The mantel test allows for a visual understanding of the relationship between community matrices and environmental factors [78]. The mantel test found that microbial communities were significantly correlated with IBA, IVA, VA, BAR, and IVAR. However, it is worth noting that the mantel test is a matrix–based method that can only detect linear relationships within the matrix and ignores nonlinear relationships. Furthermore, rumen microorganisms are highly complex and diverse, and the existence of complex interactions among them may affect the correlations. We constructed genus level correlation network diagrams to further demonstrate the existence of significant correlations among microbial communities. Therefore, insignificant correlations with other fermentation related correlations were observed, which may still play an important function in regulating the fermentation process [9,79].

LEfSe analysis identified 34 biomarkers in the rumen to elucidate key differences in the microbiota of different hybrid progeny. Of interest, rumen microbial differences may influence host phenotypes [80]. Correlation analysis of differential markers with rumen fermentation parameters revealed that their markers significantly affected AA, PA, IBA, BA, IVA, VA, TVFAs, and pH. In addition, it also significantly affected PAR, IBAR, BAR, IVAR, and VAR, but did not have a significant effect on AAR and A/P. The results of this experiment further validate that there is a robust link between differential biomarkers and the phenotype of the host [9,60]. Our analysis of microbial communities, intergroup differential markers, and rumen digestive enzymes revealed that the communities overall did not significantly correlate with digestive enzymes. On the second basis, subsequently, in our analysis of differential biomarkers with rumen enzyme activity, we found no significant correlation with enzyme activity, which is consistent with community and enzyme activity correlations. MCC is a multi–enzyme complex capable of synergistically hydrolyzing crystalline cellulose powder substrates to produce glucose, and its activity reflects a variety of enzyme activities, including endo– and exoglucanases and cellobiose glycosidases, which were significantly positively correlated at the genus level with *Prevotella* and *[Eubacterium]_ventriosum_group*, but were significantly negatively correlated with *Fretibacterium* and *Quinella*. This further confirms the consistency of the results that *Quinella* was significantly higher in rumen fluid from CAH and CDH than from Hu, while MCC was significantly lower and not significantly different among crossbred sheep. Previous studies found that increased abundance of *Quinella* correlates with decreased methane production in sheep and contributes to increased relative production of propionic acid through fermentation to form lactic acid, acetic acid plus propionic acid, or glucose hydrogen metabolism to form propionic acid [81]. However, propionic acid was not increased in this experiment. Furthermore, our study found that the biomarkers had similar correlations and their molar proportions with AA and VA. However, their correlation with PA, IBA and BA and their molar proportions were significantly different. Meanwhile, the correlation of the biomarker with TVFAs was similar to that of AA. Therefore, the VFAs produced by rumen fermentation and their molar proportion are not only influenced by the rumen microbial, but also a combination of the results of complex metabolic regulation by the organism, such as the involvement in maintaining glucose homeostasis, regulating appetite, and controlling lipid metabolism and liver regeneration [10,82].

Predictions of the microbiome indicate that rumen microbes are mainly involved in metabolic pathways, mainly including carbohydrate, amino acid, cofactors and vitamins, energy, and nucleotide metabolism pathways. Among the metabolic pathways, carbohydrate is the most abundant, which regulates carbon partitioning and sugar synthesis and conversion. Notably, in the rumen of ruminants, Bacteroidota and Firmicutes are dominant species that aid in the digestion of complex carbohydrates [12,67]. Therefore, this finding supports the observation that Bacteroidota and Firmicutes are dominant in the rumen. In addition, the rumen amino acid metabolic pathway was significantly enriched. It has been shown that rumen microorganisms with high levels of ammonia assimilation and amino acid metabolism are also involved in nitrogen metabolism, which can be used as a source of nitrogen for protein synthesis [83]. This was further corroborated with a result of previous studies that found crossbred offspring were able to improve amino acid and crude protein levels in muscle [38]. Further analysis of the top 20 pathways for microbial functional abundance prediction revealed that the Hu lambs were mainly enriched in the cysteine and methionine metabolism, amino sugar and nucleotide sugar metabolism, and biosynthesis of secondary metabolites pathways, but the hybrid progeny were mainly enriched in the microbial metabolism in diverse environments, Quorum sensing pathways, carbon metabolism, and pyruvate metabolism. Notably, pyruvate metabolism is a central part of carbon homeostasis. Predictions of microbial function revealed that enrichment of the carbon metabolism and pyruvate metabolism pathway in the hybrid progeny, which play an important function in the energy supply of rumen microorganisms, may also improve ruminant growth performance and maintain host health [84]. Therefore, in this experiment, the VFA molar concentration in rumen fluid of hybrid sheep found in this study decreased, but the VFA molar ratio remained unchanged, which may be closely related to the carbon metabolism and pyruvate metabolism pathways of rumen microbial functions of hybrid lambs. Further work is required to clarify these assumptions.

## 5. Conclusions

The present study concluded that under the same environmental and feeding conditions, the hybrid progeny significantly increased body weight and serum LDH and CRE content in lambs, but had no effect on serum biochemistry (GOT, GPT, albumin, BCA protein, GLU, HDL–C, LDL–C, TG, T–CHO, CK, and PFK), immunity (IgA, IgM, and IgG), antioxidant indices (MDA, T–AOC, CAT, GSH–Px, and T–SOD), and VFA molar proportion (acetic acid, propionic acid, butyric acid, isovaleric acid and valeric acid) which were not significantly affected. In addition, the hybrid progeny were able to affect significantly the rumen fermentation parameters, some of the fibrolytic enzymes, and the relative abundance of rumen microbial communities, increasing Verrucomicrobiota and Synergistota, which helped to enhance the organism’s body resistance as well as improve cellulose degradation and conversion to VFA for organism utilization. Correlation analysis showed that *Prevotella* had the highest percentage, while *Rikenellaceae_RC9_gut_group* was most closely correlated with other genera. The rumen microbial community was significantly correlated with VFA molar concentration and proportion. In addition, the correlations between biomarkers on VFA molar concentration and proportion were significantly different, but they were not significantly correlated with digestive enzymes. These results suggest that Charolais as a terminal sire can improve lamb production performance by influencing the structure and fermentation characteristics of microbial communities.

## Figures and Tables

**Table 1:** Effects of crossbred offspring on body size indices in sheep (*n* = 11).

Items		Body Weight, kg		Chest Circumference, cm		Body Height, cm		Body Length, cm		Tube Circumference, cm	
		0 d	135 d	0 d	135 d	0 d	135 d	0 d	135 d	0 d	135 d
H		3.83 ±0.19	29.80 ±1.30 ^b^	34.31 ±0.61	65.83 ±1.03	39.54 ±0.60	64.45 ±0.72	30.46 ±0.58	62.74· ±1.01	5.62· ±0.12	7.79 ±0.11
CAH		4.30 ±0.31	35.66 ±1.06 ^a^	35.28 ±1.01	69.64 ±1.09	39.11 ±0.86	64.08 ±1.12	28.31· ±0.77	62.28· ±1.36	6.15 ±0.22	8.70 ±0.12
CDH		3.91 ±0.22	33.67 ±0.82 ^a^	34.51 ±0.67	68.35 ±0.72	38.15 ±0.75	62.72 ±0.60	29.18· ±1.01	62.42· ±1.14	5.86 ±0.16	8.42 ±0.09
Group	H	16.81 ± 2.91		50.07 ± 3.49		51.99 ± 2.76		46.60 ± 3.57		6.70 ± 0.25	
	CAH	19.98 ± 3.46		52.46 ± 3.82		51.60 ± 2.81		45.30 ± 3.78		7.42 ± 0.30	
	CDH	18.79 ± 3.27		51.43 ± 3.72		50.43 ± 2.72		45.80 ± 3.70		7.14 ± 0.29	
*p* Value	Time	<0.001		<0.001		<0.001		<0.001		<0.001	
	Group	0.003		0.083		0.242		0.463		<0.001	
	Time × Group	0.002		0.106		0.061		0.685		0.216	

**Table 2:** Effects of crossbred offspring on serum biochemical indices in sheep (*n* = 11).

Items	H	CAH	CDH	*p* Value
GOT,U/L	12.17 ± 1.84	8.35 ± 1.34	8.93 ± 1.07	0.150
GPT, U/L	6.08 ± 0.65	3.97 ± 0.6	5.86 ± 0.67	0.051
LDH, U/L	784.76 ± 40.18 ^b^	1107.62 ± 33.36 ^a^	1045.36 ± 34.95 ^a^	<0.001
UN, mmol/L	3.32 ± 1.04 ^b^	10.15 ± 2.12 ^a^	7.08 ± 0.28 ^ab^	0.005
Albumin, g/L	18.66 ± 1.59	16.46 ± 1.27	15.37 ± 0.71	0.180
BCA protein, μg/μL	45.86 ± 2.18	40.56 ± 2.12	42.58 ± 2.05	0.219
GLU, mmol/L	2.06 ± 0.08	2.50 ± 0.26	2.29 ± 0.26	0.371
HDL–C, mmol/L	2.93 ± 0.36	2.48 ± 0.35	2.33 ± 0.31	0.438
LDL–C, mmol/L	2.09 ± 0.15	2.51 ± 0.23	2.19 ± 0.24	0.335
CRE, μmol/L	55.29 ± 2.36 ^b^	74.97 ± 3.18 ^a^	76.33 ± 2.89 ^a^	<0.001
TG, mmol/L	0.38 ± 0.05	0.43 ± 0.05	0.35 ± 0.04	0.460
T–CHO, mmol/L	3.28 ± 0.23	3.45 ± 0.25	4.52 ± 0.82	0.198
CK, U/mL	1.97 ± 0.07	1.67 ± 0.10	1.63 ± 0.21	0.188
PFK, U/mL	33.56 ± 4.32	27.26 ± 3.41	21.67 ± 2.07	0.060

Abbreviations: GOT: glutamic oxalacetic transaminase, GPT: glutamic pyruvic transaminase, LDH: lactate dehydrogenase, UN: urea nitrogen, GLU: glucose, HDL–C: high density lipoprotein cholesterol, LDL–C: low density lipoprotein cholesterol, CRE: creatinine, TG: triglyceride, T–CHO: total cholesterol, CK: creatine kinase, PFK: phosphofructokinase. In the same row, values with no letter or the same letter superscripts mean no significant difference (*p* > 0.05), while with different small letter superscripts mean significant difference (*p* < 0.05).

**Table 3:** Effects of crossbred offspring on serum immune and antioxidant indices in sheep (*n* = 11).

Items	H	CAH	CDH	*p* Value
IgA, μg/mL	35.08 ± 1.44	35.27 ± 1.49	35.87 ± 1.93	0.939
IgM, μg/mL	18.16 ± 0.63	18.63 ± 0.68	18.35 ± 1.37	0.939
IgG, μg/mL	1334.46 ± 69.45	1257.74 ± 59.32	1348.03 ± 68.20	0.584
MDA, nmol/mL	8.29 ± 0.39	7.84 ± 0.27	8.49 ± 0.31	0.366
T–AOC, U/mL	3.16 ± 0.07	3.12 ± 0.23	3.15 ± 0.24	0.987
CAT, ng/L	15.06 ± 0.38	15.21 ± 0.32	15.66 ± 0.33	0.452
GSH–Px, ng/L	11.64 ± 0.37	11.67 ± 0.22	11.73 ± 0.36	0.977
T–SOD, pg/mL	7.56 ± 0.31	7.57 ± 0.27	8.15 ± 0.31	0.297

**Table 4:** Effects of crossbred offspring on the rumen digestive enzyme activities in sheep (*n* = 11).

Items	H	CAH	CDH	*p* Value
MCC, pg/mL	112.00 ± 2.57 ^a^	94.25 ± 3.32 ^b^	98.00 ± 3.39 ^b^	0.001
β–glucosidase, ng/L	960.73 ± 27.85	996.05 ± 40.21	962.21 ± 60.23	0.831
xylanase, pg/mL	167.96 ± 3.43 ^a^	150.64 ± 5.85 ^b^	174.26 ± 5.06 ^a^	0.005
lipase, ng/mL	254.31 ± 12.92 ^a^	221.75 ± 12.35 ^b^	193.00 ± 7.48 ^b^	0.002
amylase, umol/L	150.47 ± 4.41	146.49 ± 4.37	152.05 ± 3.84	0.631
CMC, pg/mL	268.32 ± 10.14 ^a^	223.83 ± 11.56 ^b^	267.32 ± 13.24 ^a^	0.019
pepsin, ug/L	17.85 ± 0.84	16.86 ± 0.34	16.14 ± 0.67	0.190

Abbreviations: MCC: microcrystalline cellulose; CMC: carboxymethyl cellulose. In the same row, values with no letter or the same letter superscripts mean no significant difference (*p* > 0.05), while with different small letter superscripts mean significant difference (*p* < 0.05).

**Table 5:** Effects of crossbred offspring on the rumen fermentation parameters in sheep (*n* = 11).

Items	H	CAH	CDH	*p* Value
pH	6.97 ± 0.05 ^b^	7.41 ± 0.08 ^a^	7.25 ± 0.05 ^a^	<0.001
molar concentration (mmol/L)				
acetic acid	64.81 ± 5.56 ^a^	44.81 ± 6.08 ^b^	38.38 ± 2.51 ^b^	0.002
propionic acid	18.18 ± 0.54 ^a^	11.45 ± 1.03 ^b^	13.29 ± 0.45 ^b^	<0.001
isobutyric acid	1.34 ± 0.05 ^a^	1.08 ± 0.08 ^b^	1.25 ± 0.05 ^ab^	0.021
butyric acid	13.88 ± 0.62 ^a^	9.80 ± 0.82 ^b^	10.21 ± 0.72 ^b^	0.001
isovaleric acid	1.77 ± 0.47	1.18 ± 0.11	1.47 ± 0.07	0.351
valeric acid	1.08 ± 0.11 ^a^	0.58 ± 0.05 ^b^	0.67 ± 0.03 ^b^	<0.001
TVFAs	101.06 ± 5.46 ^a^	68.91 ± 7.57 ^b^	65.28 ± 3.39 ^b^	<0.001
acetic acid/propionic acid	3.64 ± 0.38	3.93 ± 0.37	2.87 ± 0.13	0.067
molar proportion				
acetic acid	63.11 ± 2.01	63.66 ± 1.92	58.38 ± 0.95	0.069
propionic acid	18.61 ± 1.27	17.18 ± 1.06	20.65 ± 0.75	0.080
isobutyric acid	1.35 ± 0.06 ^c^	1.64 ± 0.10 ^b^	1.94 ± 0.07 ^a^	<0.001
butyric acid	14.11 ± 0.91	14.87 ± 0.93	15.69 ± 0.81	0.459
isovaleric acid	1.71 ± 0.39	1.78 ± 0.10	2.30 ± 0.13	0.193
valeric acid	1.11 ± 0.16	0.87 ± 0.04	1.04 ± 0.05	0.210

In the same row, values with no letter or the same letter superscripts mean no significant difference (*p* > 0.05), while with different small letter superscripts mean significant difference (*p* < 0.05).

**Table 6:** Effects of crossbred offspring on the rumen microorganisms alpha diversity (*n* = 11).

Items	H	CAH	CDH	*p* Value		
				H:CAH	H:CDH	CAH:CDH
ACE index	875.73 ± 44.20 ^b^	1104.71 ± 22.72 ^a^	1069.16 ± 50.03 ^a^	<0.001	0.005	0.560
Chao1 index	873.60 ± 44.06 ^b^	1101.61 ± 22.80 ^a^	1066.98 ± 49.82 ^a^	<0.001	0.005	0.610
Simpson index	9.90 × 10^−1^ ± 0.00	9.93 × 10^−1^ ± 0.00	9.91 × 10^−1^ ± 0.00	0.300	0.056	0.480
Shannon index	8.16 ± 0.17	8.44 ± 0.11	8.52 ± 0.15	0.920	0.130	0.120

In the same row, values with no letter or the same letter superscripts mean no significant difference (*p* > 0.05), while with different small letter superscripts mean significant difference (*p* < 0.05).

**Table 7:** Microbial functional KEGG enrichment analysis of crossbred offspring and Hu sheep.

Items			H	CAH	CDH	*p* Value		
Class 1	Class 2	Class 3				H: CAH	H: CDH	CAH: CDH
ME	Amino acid metabolism	Cysteine and methionine metabolism	9.92 × 10^−3^	9.74 × 10^−3^	9.81 × 10^−3^	0.035	0.289	1.101
		Alanine, aspartate and glutamate metabolism	9.80 × 10^−3^	9.71 × 10^−3^	9.61 × 10^−3^	0.318	0.140	0.889
	Biosynthesis of other secondary metabolites	Biosynthesis of antibiotics	5.80 × 10^−2^	5.79 × 10^−2^	5.79 × 10^−2^	0.448	0.805	0.946
	Carbohydrate metabolism	Amino sugar and nucleotide sugar metabolism	1.19 × 10^−2^	1.17 × 10^−2^	1.17 × 10^−2^	0.037	0.172	1.002
		Glycolysis/Gluconeogenesis	1.07 × 10^−2^	1.07 × 10^−2^	1.07 × 10^−2^	0.719	0.786	1.017
		Pyruvate metabolism	9.59 × 10^−3^	9.79 × 10^−3^	9.73 × 10^−3^	0.022	0.204	0.947
	Energy metabolism	Oxidative phosphorylation	1.02 × 10^−2^	1.02 × 10^−2^	1.02 × 10^−2^	0.751	0.827	0.990
		Carbon fixation pathways in prokaryotes	9.55 × 10^−3^	9.71 × 10^−3^	9.68 × 10^−3^	0.318	0.578	0.992
	Global and overview maps	Metabolic pathways	1.75 × 10^−1^	1.74 × 10^−1^	1.74 × 10^−1^	0.299	0.446	0.967
		Biosynthesis of secondary metabolites	7.98 × 10^−2^	7.94 × 10^−2^	7.95 × 10^−2^	0.032	0.302	0.968
		Biosynthesis of amino acids	4.06 × 10^−2^	4.03 × 10^−2^	4.02 × 10^−2^	0.461	0.444	0.979
		Microbial metabolism in diverse environments	3.71 × 10^−2^	3.76 × 10^−2^	3.75 × 10^−2^	0.030	0.176	0.952
		Carbon metabolism	2.63 × 10^−2^	2.65 × 10^−2^	2.64 × 10^−2^	0.033	0.550	0.982
	Nucleotide metabolism	Purine metabolism	2.16 × 10^−2^	2.15 × 10^−2^	2.16 × 10^−2^	0.171	0.581	1.032
		Pyrimidine metabolism	1.87 × 10^−2^	1.86 × 10^−2^	1.85 × 10^−2^	0.476	0.460	1.014
GIP	Translation	Ribosome	2.59 × 10^−2^	2.57 × 10^−2^	2.59 × 10^−2^	0.332	1.000	0.957
		Aminoacyl–tRNA biosynthesis	1.09 × 10^−2^	1.09 × 10^−2^	1.10 × 10^−2^	0.709	0.461	0.951
EIP	Membrane transport	ABC transporters	2.16 × 10^−2^	2.21 × 10^−2^	2.18 × 10^−2^	0.366	0.711	0.967
	Signal transduction	Two–component system	1.71 × 10^−2^	1.72 × 10^−2^	1.72 × 10^−2^	0.642	0.697	0.997
CPr	Cellular community–prokaryotes	Quorum sensing	1.13 × 10^−2^	1.17 × 10^−2^	1.15 × 10^−2^	0.040	0.176	0.945

Abbreviations: ME: Metabolism, GIP: Genetic Information Processing, CPr: Cellular Processes, EIP: Environmental Information Processing.

## Data Availability

Follow–up research on this project is ongoing; please contact the corresponding author with reasonable requests.

## Supplementary Materials

The following supporting information can be downloaded at: https://www.mdpi.com/article/10.3390/ani14101509/s1, Supplementary Table S1 Quality assessment of sequencing data; Supplementary Figure S1. Figure S1a:ASVs–Venn diagram analysis of H, CAH and CDH, Different ellipse colors represent different groups, the overlapping part represents the number of Features common to the group, and the non–overlapping part is the number of Features specific to the group; Figure S1b:Dilution curve analysis, Different colored lines in the upper right corner represent different subgroups, the horizontal coordinate is the amount of randomly selected sequencing data, and the vertical coordinate is the number of observed features; Effect of crossbred offspring on the relative abundance of the rumen microbial at phylum level Top 10 (%), Supplementary Table S2; Effect of crossbred offspring on the relative abundance of the rumen microbial at genus level Top 10 (%), Supplementary Table S3.
