# Supplementary material for: Relationship between Rumen Microbial Differences and Phenotype Traits among Hu Sheep and Crossbred Offspring Sheep
Source: Animals (Basel). 2024 May 20;14(10):1509. doi: 10.3390/ani14101509 (PMC11117386; doi:10.3390/ani14101509)
Supplement: Supplementary file 1 [file animals-14-01509-s001.zip › animals-2989339-supplementary.pdf]

Relationship between rumen microbial differences and phe-notype traits among Hu sheep and Crossbred offspring sheep

supplementary Table S1 Quality assessment of sequencing data

Sample	Raw Reads	Clean Reads	Denoised Reads	Merged Reads	Non-chimeric Reads
H1	80191	79970	78763	77605	76397
H2	80026	79794	78068	76292	74791
H3	79896	79685	78264	76381	74955
H4	79932	79687	78772	77584	77051
H5	79800	79586	77874	76171	74563
H6	80004	79786	78241	76200	74129
H7	80127	79914	78127	76062	73980
H8	79775	79569	78063	76228	74398
H9	80068	79866	78607	77061	75294
H10	79851	79642	77743	75455	72813
H11	79974	79758	78038	76095	74494
CAH1	79888	79682	77968	75894	74454
CAH2	80111	79882	78162	75935	74057
CAH3	80151	79920	77974	75994	74203
CAH4	80256	80047	78022	75745	73446
CAH5	79893	79666	77611	75225	73063
CAH6	79988	79794	78007	76072	73943
CAH7	79993	79766	77317	74735	72909
CAH8	79965	79766	77581	75179	73447
CAH9	80141	79912	78093	75746	73242
CAH10	79857	79626	77849	75679	73864
CAH11	79957	79768	78165	76424	74703
CDH1	80027	79800	78105	76220	74511
CDH2	79954	79723	77677	75335	72908
CDH3	79735	79525	77640	75410	73620
CDH4	79879	79674	78336	77017	75169
CDH5	79836	79643	78364	76940	75718
CDH6	79886	79693	77240	74488	72560
CDH7	80238	80035	78468	76629	74778
CDH8	80042	79826	77660	75249	73608
CDH9	79960	79770	77543	75301	72934
CDH10	79984	79789	77907	75814	74085
CDH11	80274	80062	78336	76284	74556

Sample is the name of the sample; Raw Reads is the number of raw reads obtained from sequencing; Clean Reads is the number of high quality reads obtained after quality control of the original sequence; Denoised Reads is the number of reads after denoising the Clean Reads; Merged Reads is the number of sequence entries obtained after splicing Denoised Reads according to overlap; Non-chimeric Reads is the number of sequence entries after final chimera removal

a b

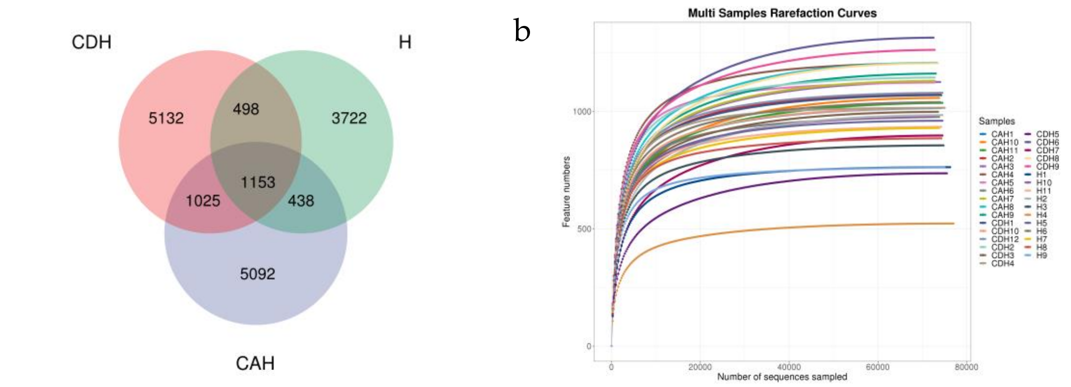

Supplementary Figure S1. Figure S1a:ASVs-Venn diagram analysis of H, CAH and CDH, Different ellipse colors represent different groups, the overlapping part represents the number of Features common to the group, and the non-overlapping part is the number of Features specific to the group ; Figure S1b:Dilution curve analysis, Different colored lines in the upper right corner represent different subgroups, the horizontal coordinate is the amount of randomly selected sequencing data, and the vertical coordinate is the number of observed features.

Supplementary Table S2 Effect of crossbred offspring on the relative abundance of the rumen microbial at phylum level Top 10 (%)

Items	H	CAH	CDH	*p* Value
Bacteroidota	53.65±6.62	50.57±3.73	50.72±5.74	0.507
Firmicutes	39.92±4.69	38.00±5.15	38.63±4.00	0.680
Proteobacteria	3.50±3.09	4.92±4.16	4.74±3.85	0.657
Patescibacteria	0.85±0.40	1.66±1.05	1.43±0.88	0.240
Spirochaetota	0.65±0.53	1.51±0.86	1.19±0.71	0.122
Synergistota	0.15±0.05	1.56±1.00	1.38±1.04	0.017
unclassified_Bacteria	0.35±0.63	0.50±0.29	0.99±0.73	0.145
Verrucomicrobiota	0.20±0.09	0.58±0.29	0.47±0.29	0.032
Desulfobacterota	0.17±0.08	0.28±0.15	0.19±0.10	0.227
Fibrobacterota	0.32±0.23	0.16±0.13	0.12±0.10	0.111

Supplementary Table S3 Effect of crossbred offspring on the relative abundance of the rumen microbial at genus level Top 10 (%)

Items	H	CAH	CDH	*p* Value
Prevotella	22.16±7.04	16.91±4.19	20.07±3.78	0.415
uncultured_rumen_bacterium	5.36±1.04	8.09±1.13	7.87±2.35	0.023
Rikenellaceae_RC9_gut_group	5.01±1.46	6.23±1.08	6.40±1.98	0.446
unclassified_Prevotellaceae	5.25±3.60	7.54±6.42	3.96±2.92	0.597
unclassified_Bacteroidales_RF16_group	5.65±2.55	4.31±1.23	4.03±1.58	0.503
unclassified_Lachnospiraceae	3.21±0.79	2.77±1.33	3.67±2.94	0.640
unclassified_F082	2.42±0.58	3.42±1.12	3.11±1.60	0.535
Succiniclasticum	3.20±0.79	3.06±1.17	2.46±1.00	0.597
Prevotellaceae_UCG_003	2.62±0.90	2.75±0.61	3.12±1.09	0.528
Ruminococcus	3.06±1.31	1.98±0.68	2.72±1.56	0.515
